# CACYBP knockdown inhibits progression of prostate cancer via p53

**DOI:** 10.1007/s00432-022-04497-x

**Published:** 2022-12-28

**Authors:** Qiang Li, Zhili Liu, Luping Ma, Weiqi Yin, Kan Zhang

**Affiliations:** 1 Department of Urological Surgery, First Affiliated Hospital School of Medicine, Shihezi University, Shihezi, 832008 Xinjiang China; 2 Department of Urological Surgery, Ningbo First Hospital, Ningbo, 315010 Zhejiang China

**Keywords:** PC, CACYBP, Proliferation, Apoptosis, Cell cycle, Migration

## Abstract

**Purpose:**

Prostate cancer (PC) is one of the most common malignant tumors of genitourinary system in men. CACYCLIN binding protein (CACYBP) is involved in the progression of a variety of cancers. The aim of this study was to explore the expression and functional role of CACYBP in PC.

**Methods:**

The expression of CACYBP in PC was evaluated by immunohistochemical (IHC) staining and qRT-PCR. Subsequently, we established lentivirus-mediated CACYBP knockdown in PC cell lines. The biological roles of CACYBP on proliferation, apoptosis, cycle distribution, migration and tumor formation of PC were investigated by Celigo cell counting assay, flow cytometry, transwell assay, wound-healing assay and mice xenograft models, respectively.

**Results:**

CACYBP was highly expressed in PC and was positively correlated with the pathological grade of PC patients. Knockdown of CACYBP inhibited proliferation, enhanced apoptosis, arrested cell cycle in G2 and suppressed migration of PC cell lines in vitro. In addition, CACYBP knockdown weakened the tumor growth of PC in vivo. Moreover, addition of p53 inhibitor could effectively alleviate the inhibitory effect of CACYBP knockdown on cell activity.

**Conclusion:**

This study revealed that knockdown of CACYBP inhibited the proliferation, migration and tumorigenicity of PC, which may serve as a potential therapeutic target for the treatment of PC.

## Introduction

Prostate cancer (PC) is the second most common male genitourinary system tumor with a high incidence, complex and multifocal features and atypical early clinical symptoms (Davidsson et al. 2018; Mehra et al. 2021). In addition, PC harbors numerous genetic and epigenetic abnormalities that drive unrestricted cellular growth and increased metabolic demands (Yadav et al. 2018). In the past few years, surgery, hormone therapy (androgen deprivation therapy), cryotherapy combined with chemotherapy and radiotherapy were the main treatments for PC (Evans 2018). Although these traditional therapies could prolong the survival of patients, they may undergo tumor metastasis, recurrence and even complications and serious side effects (Galletti et al. 2017; Nevedomskaya et al. 2018; Rosellini et al. 2021; Ruiz de Porras et al. 2021). In recent years, advances in molecular biology technology have led to the innovation of cancer treatment environment. Some cutting-edge therapies such as molecular targeted therapy and immune checkpoint blocking therapy have attracted widespread attention (Grüllich et al. 2020). In particular, targeted therapy has become a key treatment strategy for PC patients (Czerwińska et al. 2020). Importantly, the phase III study for PARP inhibitors has shown good efficacy in PC patients (Ratta et al. 2020). Nevertheless, targeted therapies for PC are extremely limited. Therefore, exploration of molecular mechanism of PC and recognition of specific molecules are crucial for the development of potential molecular therapeutic targets.

Calcyclin-binding protein (CACYBP) is a 26 kDa protein identified in Ehrlich ascites tumor (EAT) cells and binds to Calcyclin (S100A6) (Zheng and Chen 2021). CACYBP was subsequently found to bind to the E3 ubiquitinated ligase SIAH1, also known as SIAH1 interacting protein (SIP). In addition, CACYBP participates in a wide range of cellular processes, including cell differentiation, proliferation, cytoskeletal dynamics and tumorigenesis (Zheng and Chen 2021). Previous reports have shown that CACABP emerges with different expression levels in a variety of cancers such as gastric cancer (Ning et al. 2007), pancreatic cancer (Chen et al. 2011), colon cancer (Zhai et al. 2017), renal cancer (Sun et al. 2007), breast cancer (Nie et al. 2010) and hepatocellular carcinoma (Lian et al. 2019), and contributes to the progression of malignancies. Furthermore, CACYBP may play a role in tumorigenesis by participating in the degradation of cancer-related proteins (Ning et al. 2007). However, the biological function of CACYBP in PC has not been elucidated.

In this study, the expression of CACYBP in PC tissues was evaluated by immunohistochemical staining and qRT-PCR. Subsequently, the biological roles of CACYBP in PC cell were investigated by Celigo cell counting assay, flow cytometry, transwell assay and wound-healing assay. In addition, the mice xenograft model was established for subsequent verification in vivo. Herein, this study demonstrated that CACYBP was involved in the progression of PC and may be a potential therapeutic target for PC.

## Materials and methods

### Tissue collection and immunohistochemistry (IHC)

Tissue microarray of PC patients was obtained from Shanghai Outdo Biotech Company (#JS W-11-01), which included 87 tumor tissues and 45 para-carcinoma tissues. Ethical approval was obtained from the Ethics Committee of First Affiliated Hospital, School of Medicine, Shihezi University. In brief, tumor specimens were dewaxed by xylene for 15 min and rehydrated by 100% alcohol for 10 min. The antigen was repaired in 0.01 M sodium citrate buffer (pH = 6.0) at 180 ℃ for 5 min and blocked by 3% hydrogen peroxide bath for 15 min. After washing, the sections were incubated with anti-CACYBP (1: 50, Cat. # ab171972, Abcam) or anti-Ki67 (1:200, Cat. # ab16667, Abcam) overnight at 4 ℃ and followed by conjugation to the Goat Anti-Rabbit IgG H&L (HRP) antibody (1: 400, Abcam, USA, Cat. # ab6721) at room temperature for 2 h. Tissue sections were stained with DAB and hematoxylin, and then exanimated with microscopic (Olympus). All specimens were classified into categories based on staining percentage and staining intensity as previously described (Feng et al. 2021).

### Cell lines and cell culture

Human PC cell lines LNCaP, DU145, PC-3 and the normal prostate stromal cell line WPMY were purchased from Shanghai Genechem Co., Ltd (Shanghai, China). All the cell lines were maintained in a humidified atmosphere containing 5% CO_2_ at 37 ℃. Specifically, LNCaP, DU145 and WPMY cells were cultured in RPMI 1640 (Corning) supplemented with 10% FBS (Invitrogen, Carlsbad, CA, USA), whereas PC-3 cells were cultured in F-12K (Corning) containing 10% FBS (Invitrogen, Carlsbad, CA, USA).

### Cell transfection

Small-interfering RNA (siRNA) oligos targeting CACYBP (shCACYBP) and a siRNA with a non-targeting sequence (scrambled sequence as negative control, shCtrl) were synthesized (Bioscienceres, Shanghai, China). The RNA interference target sequences (shCtrl: 5′-TTCTCCGAACGTGTCACGT-3′; shCACYBP: 5′-AGCCAAAGGAGACACGGAATT-3′, 5′-ATGATATGAAGCGAACCATTA-3′, 5′-GAATCTAAATGGGAAGAGTTA-3′) were designed and cloned into BR-V-108 vectors (Bioscienceres, Shanghai, China) using T4 DNA ligase. The recombinant lentiviral vector was transfected into 293 T cells and extracted the plasmid (EndoFree maxi plasmid kit, Tiangen, Beijing, China). Subsequently, LNCaP and DU145 cells were infected with the recombinant lentiviral containing the corresponding sequences (shCtrl or shCACYBP) at 37 ℃ using Lipofectamine^®^ 3000 (Invitrogen). After cultured for 72 h, infection efficiency was evaluated under a fluorescence microscope (200× magnification, OLYMPUS).

### Quantitative real time PCR (qRT-PCR)

Total RNA was isolated from LNCaP and DU145 cells using TRIzol^®^ reagent (Sigma, USA). The concentration of RNA was assessed by Nanodrop 2000/2000C spectrophotometry (Thermo Fisher Scientific). Subsequently, high-quality cDNA was synthesized with SuperScript first strand synthesis system (Thermo Fisher Scientific, USA) according to the manufacturer’s instructions. Then, qRT-PCR was performed by the ${2}^{{ - \Delta \Delta C_{{\text{t}}} }}$ method using the AceQ qPCR SYBR Green master mix (Vazyme, Nanjing, China). The primer sequences were used as follows: CACYBP, forward: 5′-ACAGATCCTAGTGAGGGATTGATG-3′ and reverse: 5′-TCCGTGTCTCCTTTGGCTTG-3′; GAPDH (reference control): forward: 5′-TGACTTCAACAGCGACACCCA-3′ and reverse: 5′-CACCCTGTTGCTGTAGCCAAA-3′.

### Western blotting analysis

LNCaP and DU145 cells were lysed with ice-cold RIPA lysis buffer (Millipore) and total protein was collected. Subsequently, protein concentration was detected by a BCA Protein Assay Kit (HyClone-Pierce). Equal amount proteins (20 µg) were separated by 10% SDS-PAGE (Invitrogen) and transferred to PVDF membrane (BIO-RAD). The membrane was blocked in TBST solution containing 5% non-fat milk for 1 h and incubated with primary antibodies (CACYBP antibody, 1:3000, Cat. #ab171972, Abcam; p53 antibody, 1:2000, Cat. #10442-1-AP, Proteintech; p-p53 antibody, 1:2000, Cat. #28961-1-AP, Proteintech; Bax antibody, 1:2000, Cat. #ab182733, Abcam; Bcl-2 antibody, 1:2000, Cat. #ab182858, Abcam; GAPDH antibody, 1:3000, Cat. #60004-1-lg, Proteintech) at room temperature for 2 h. Then, the membrane was continuingly incubated with the secondary antibody Goat Anti-Rabbit (1:3000, Cat. #A0208, Beyotime) at room temperature for 1 h. Protein bands were visualized by ECL plus TM Western blotting system kit (Millipore).

### Celigo cell counting assay

The numbers of LNCaP and DU145 cells were assessed by performing Celigo cell counting assay. Cells were seeded into 96-well plates at a density of 2000 cells per well in triplicate. After culturing in an incubator containing 5% CO_2_ at 37 ℃ for 24 h, Celigo image cytometer (Nexcelom Bioscience, Lawrence, MA, USA) was utilized to capture cell images and quantify cell numbers once a day for 5 days. Cell proliferation curves were drawn for each group.

### CCK-8 assay

DU145 cells were seeded into a 96-well plate with a cell density of 2000 cells per well and further cultured with a specific p53 inhibitor Pifithrin-α (PFTα, 1:2000, Cat. # 28961-1-AP, Proteintech) or not at 37 ℃. Ten µL CCK-8 solution (Sigma, USA) was added into the wells and incubated for 4 h, the absorbance at 450 nm was measured with a microplate reader (Tecan infinite). Each assay was conducted in triplicate and the cell growth curve was created.

### Flow cytometry

Apoptosis and cell cycle of LNCaP and DU145 cells were examined by flow cytometry. Briefly, the cells were seeded into 6 cm dish at a seeding density of 1 × 10^3^ cells/well in triplicate and cultured for 5 days. Further, cells were trypsinized, washed with PBS (pH = 7.2–7.4) and centrifuged (4 ℃, 3000*g*, 10 min). For cell apoptosis assay, cells were resuspended with 500 µL diluted 1× binding buffer (eBioscience, Thermo Fisher Scientific) and stained with 10 µL Annexin V-APC in the dark for 10 min at room temperature. The percentage of cell apoptotic rate was measured by FACSCalibur (BD Biosciences). For cell cycle assay, cells were fixed with cold ethanol (70%) for 1 h at 4 ℃ and stained by propidium iodide (PI) (40×, 2 mg/mL: 100× RNase, 10 mg/mL: 1× PBS = 25:10:1000) for 30 min at room temperature. Subsequently, cell cycle distribution (G1, S and G2) was detected by FACSCalibur and analyzed with Flow Jo software (version vX 0.7) (BD Franklin Lakes, USA).

### Wound-healing assay

LNCaP and DU145 cells with or without CACYBP knockdown were seeded into 96-well plats. After cell growing for 24 h, the complete medium was changed into medium with lower serum concentration. Then, we scratched a wound across the cell layer using a 96-wounding replicator (Cat. #VP408FH, VP scientific). Cell debris were slightly rinsed with serum-free medium for 2–3 times. Images were captured at 0, 24 and 48 h by a fluorescence microscope (50× magnification, OLYMPUS). Cell migration rate of each group was calculated based on the migration distance.

### Transwell assay

LNCaP and DU145 cells were digested with trypsin and resuspended with lower serum concentration. 100 µL cells suspension (total 5 × 10^5^ cells) was loaded into the upper chamber of transwell, while 600 µL culture medium supplemented with 30% FBS was added to the lower chamber. After incubation 24 h at 37 °C, the migratory cells on the lower surface of membrane were fixed with 4% paraformaldehyde and stained with 0.1% of crystal violet at room temperature. Following washing with PBS, five fields of view per well were selected randomly under a fluorescence microscope (200× magnification, OLYMPUS), and the migration rate was calculated according to number of migratory cells.

### Mice xenograft model

Female BALB/c nude mice (4-week-old) were obtained from Beijing Vital River Laboratory Animal Technology Co., Ltd. All mice were kept in specific pathogen-free (SPF) facilities at 22 ± 3 ℃, 55 ± 5% humidity under a 12 h light/dark cycle from 8:00 to 20:00, and had free access to water and food. All the experimental protocol of these mice was approved by Ethics Committee of First Affiliated Hospital, School of Medicine, Shihezi University. Mice were randomly divided into two groups (shCACYBP *vs.* shCtrl groups, *n* = 10 for each group) and subcutaneously injected with 0.2 mL lentivirus-transfected DU145 cell suspensions. After 35 days of the construction of xenograft models, tumor growth was monitored and tumor volume was calculated every two days according to the formula: *π*/6 × length × width^2^. On the day of the last measurement, the tumors were removed and subjected to weighing.

### Human phospho-kinase array

The relative levels of protein phosphorylation in DU145 cells with or without CACYBP knockdown were detected using the human phospho-kinase array Kit (ARY003C, Bio-Techne, China). Briefly, membranes were blocked in array buffer for 1 h at room temperature, maintained with cell lysates overnight at 4 °C, and incubated with antibody cocktails for 2 h at room temperature. Membranes were washed and incubated with array buffer containing diluted streptavidin–horseradish peroxidase (HRP) for 30 min. Each spot corresponding to the amount of phosphorylated protein bound was acquired using enhanced ECL (Amersham). Signal densities were quantitated using Quantity One software (National Institute of Health) and normalized to the α-tubulin levels.

### Statistical analysis

Statistical analyses were performed using SPSS 20.0 (SPSS, Chicago, IL, USA) and GraphPad prism 6.0 (GraphPad, La Jolla, CA, USA). Student’s *t* test and Chi-square test were used during the data analysis. One-way analysis of variance test was used for the comparison of the significant differences for multiple groups. ${2}^{{ - \Delta \Delta C_{{\text{t}}} }}$ method was used during the qPCR assays. Data are presented as the mean ± standard deviations (SD). *P* value < 0.05 was considered as statistically significant difference.

## Results

### CACYBP was overexpressed in PC

**Fig. 1:**
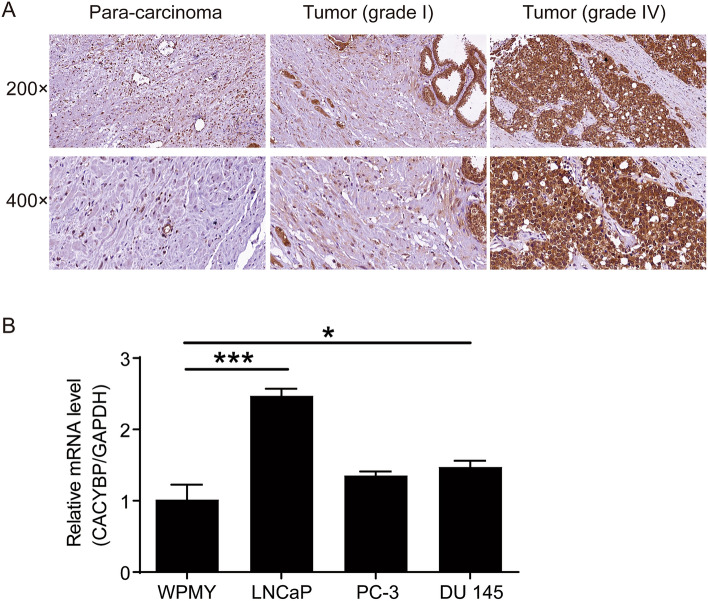
Expression of CACYBP in PC. **A** Negative staining in adjacent normal tissues and representative immunohistological characteristics with high expression of CACYBP in PC tissues. **B** The background expression of CACYBP in PC cell lines and normal human prostate stromal cell line WPMY was detected by qCPR

In order to clarify the role of CACYBP in the development and progression of PC, the expression of CACYBP in 87 tumor tissues and 45 para-carcinoma tissues was detected by IHC analysis. The results revealed that high expression of CACYBP was mainly found in the PC, while low expression was present in para-carcinoma tissues (Table 1; Fig. 1A). Furthermore, the tumor tissue of patients with advanced PC was more abundant in CACYBP than that of patients with early PC (Fig. 1A; Table 1), suggesting that the expression of CACYBP in PC was positively correlated with pathological grade. In addition, the mRNA levels of CACYBP in 3 PC cell lines DU145, LNCaP, PC-3 and the normal prostate stromal cell line WPMY were detected by qRT-PCR. As shown in Fig. 1B, CACYBP was highly expressed in LNCaP and DU145 cells (*P* < 0.05) compared with WPMY. These results demonstrated that CACYBP was highly expressed in PC and was positively correlated with the pathological grade of PC patients.

**Table 1:** Relationship between CACYBP expression and tumor characteristics in patients with prostate cancer

Features	No. of patients	CACYBP expression		*P* value
		Low	High	
All patients	87	50	37	
Age (years)	88	37	51	0.369
Gleason score	87	36	51	0.117
Grade				0.014*
1	19	13	6	
2	38	14	24	
3	18	3	15	
4	4	1	3	
5	5	3	2	
PDL1 serosa carcinoma expression	83	33	50	0.822

*P value < 0.05

### Establishment of CACYBP knockdown in PC cell lines

**Fig. 2:**
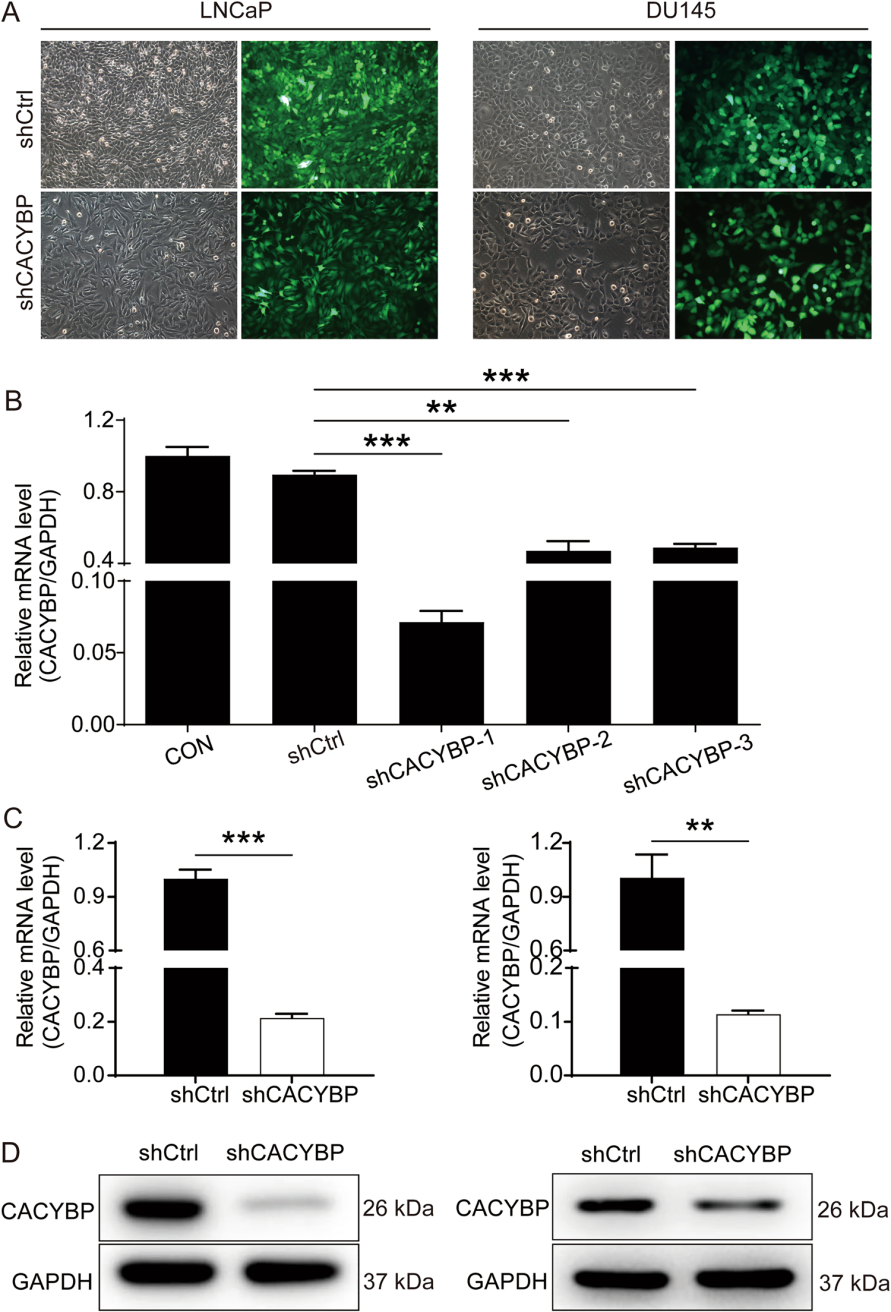
Construction of PC cell models with CACYBP knockdown. **A** The fluorescence of LNCaP and DU145 cells was observed and used to represent the transfection efficiency of shCACYBP and shCtrl. **B** The efficiency of 3 shRNAs targeting CACYBP was evaluated by qCPR. **C**, **D** The knockdown efficiency of CACYBP in LNCaP and DU145 cells was evaluated by qPCR (**C**) and further verified by western blotting (**D**). The representative images were randomly selected from at least three independent experiments in duplicate. **P < 0.01, ***P < 0.001

CACYBP-shRNA lentivirus or shCtrl lentivirus (as negative control) were transfected into LNCaP and DU145 cell lines to establish the CACYBP knockdown cell models. After cell transfection with lentivirus, a large amount of green fluorescent protein could be observed under fluorescence field, suggesting that the cell transfection was successful (Fig. 2A). Subsequently, we tested the mRNA levels of CACYBP in LNCaP cell line with CACYBP knockdown using qRT-PCR. These results showed that the mRNA expression of CACYBP in shCACYBP-1, shCACYBP-2 and shCACYBP-3 cells was decreased compared with the shCtrl cells (Fig. 2B). The knockdown efficiency of CACYBP in shCACYBP-1 cells was higher than shCACYBP-2 and shCACYBP-3 cells (*P* < 0.001); thus, the shCACYBP-1 was utilized in subsequent experiments. Compared with shCtrl group, the mRNA level of CACYBP was reduced by 78.61% in LNCaP cells and 88.67% in DU145 cells, respectively (*P* < 0.001(Fig. 2C)). Western blotting assay demonstrated that the protein expression of CACYBP in shCACYBP group was down-regulated as compared with shCtrl group (Fig. 2D). Thus, CACYBP knockdown cell model was successfully constructed for the subsequent experiments.

### CACYBP knockdown suppressed the proliferation and enhanced apoptosis of PC cell lines

**Fig. 3:**
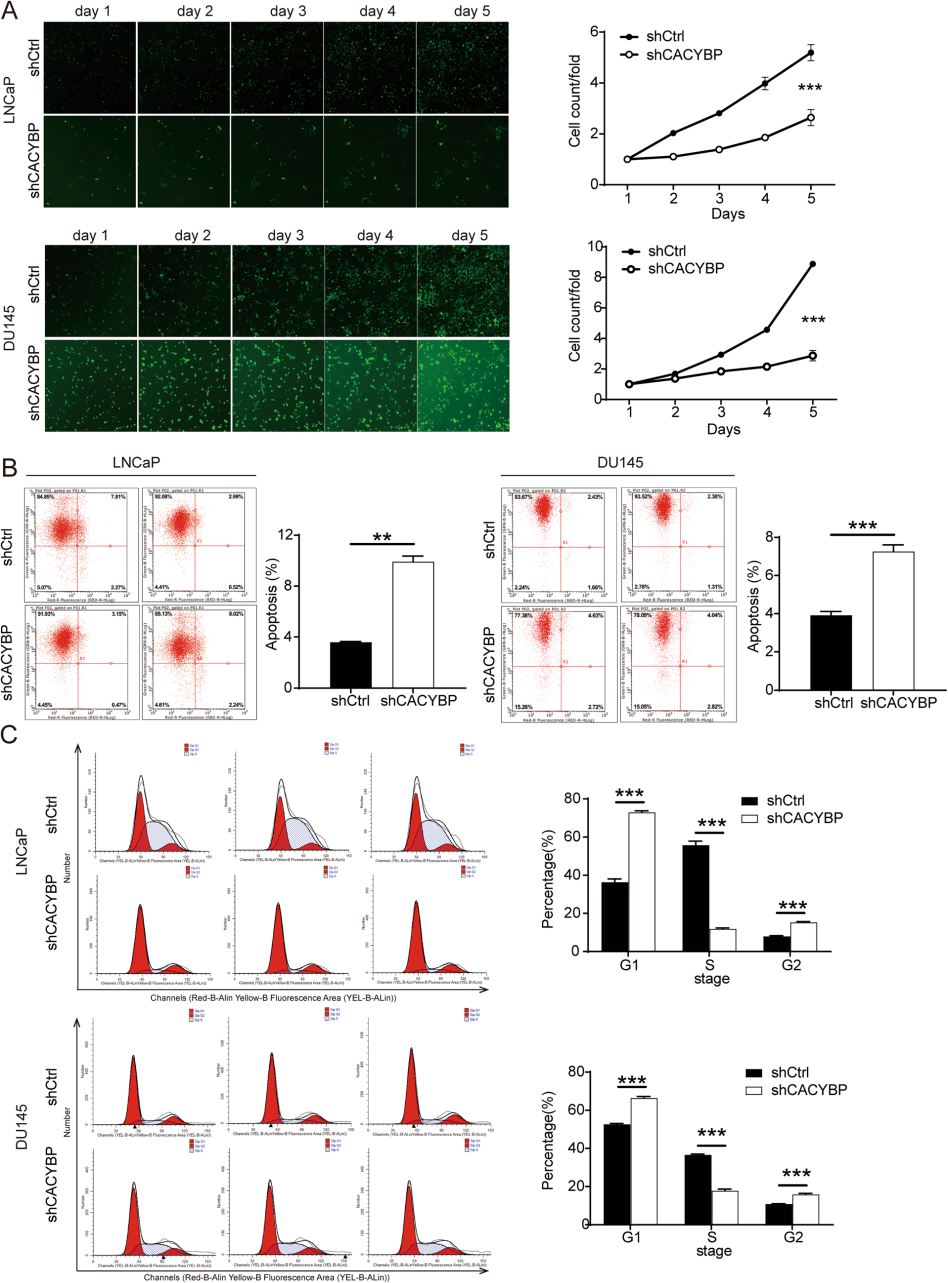
CACYBP knockdown inhibited proliferation of LNCaP and DU145 cells and blocked the cell cycle. **A** The effect of CACYBP knockdown on the viability of LNCaP and DU145 cells was investigated by Celigo cell counting assay. **B**, **C** The effect of CACYBP knockdown on cell apoptosis (**B**) and cell cycle (**C**) of LNCaP and DU145 cells was analyzed by flow cytometry. **P < 0.01, ***P < 0.001

The effect of CACYBP on the functions of PC cells was evaluated. Celigo assay was performed to explore the effect of CACYBP on cell growth in LNCaP and DU145 cell lines. As illustrated in Fig. 3A, the proliferation of cells was significantly inhibited in shCACYBP group relative to the shCtrl group (*P* < 0.001). Moreover, the effects of CACYBP on apoptosis and cell cycle detection were assessed in LNCaP and DU145 cells by flow cytometry. As shown in Fig. 3B, the cell apoptosis percentage was significantly increased in shCACYBP group compared with shCtrl group. These results suggested that CACYBP knockdown notably enhanced cell apoptosis in PC cell lines. Furthermore, cell cycle analysis showed that the proportion of the cells in the G2 phase increased and the proportion of cells in the S phase decreased of the shCACYBP group (Fig. 3C). These results revealed that CACYBP knockdown inhibited the cell proliferation, induced cell apoptosis and promoted G2-arrest of PC cells.

### CACYBP knockdown inhibited the migration of PC cells

**Fig. 4:**
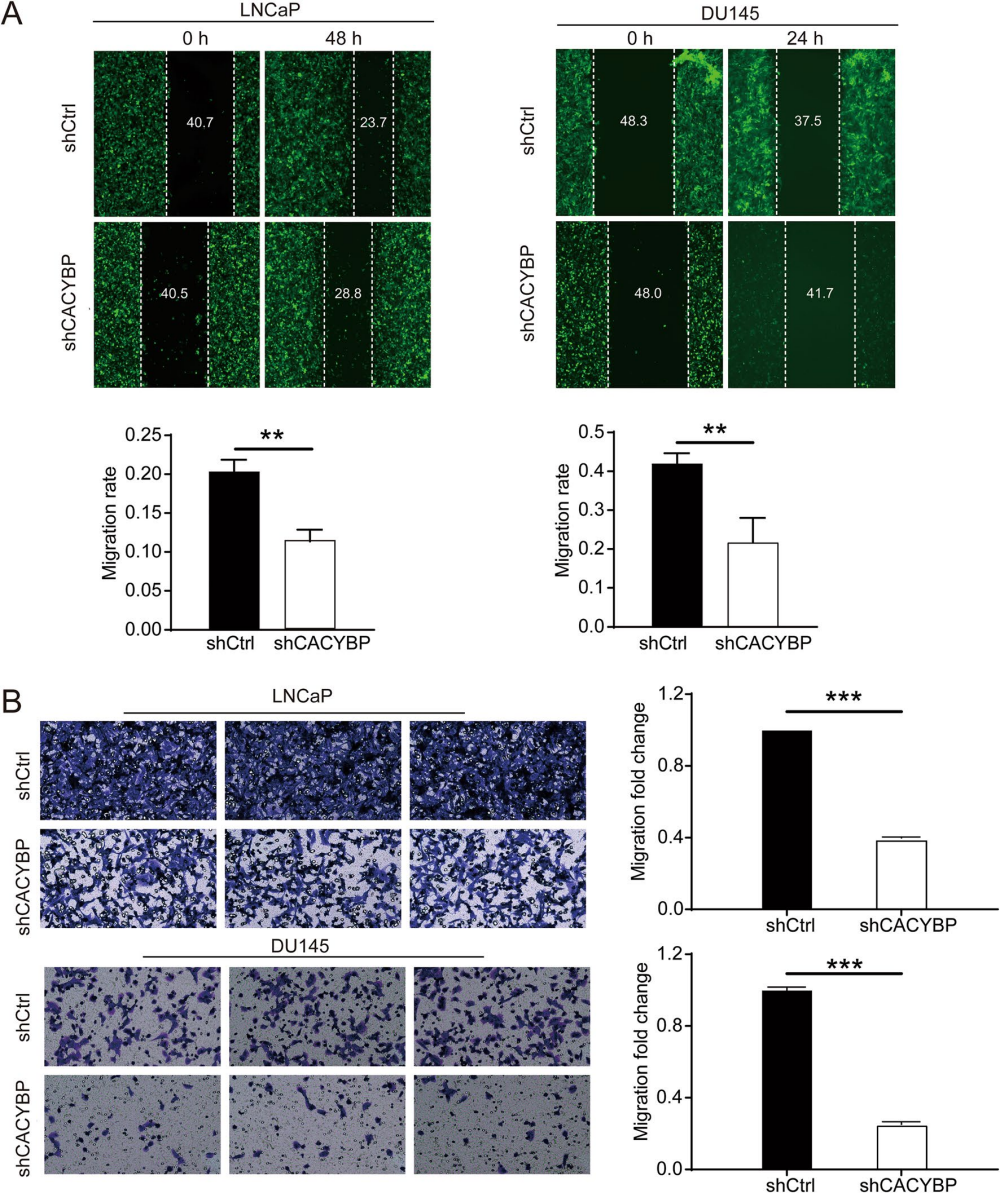
CACYBP knockdown inhibited the migration of LNCaP and DU145 cells. **A** Cell migration of LNCaP and DU145 cells with or without knockdown of CACYBP was evaluated by wound-healing assay. **B** Cell migration invasion of LNCaP and DU145 cells with or without knockdown of CACYBP was evaluated by transwell assay. The data were expressed as mean ± SD (*n* = 3), **P < 0.01, ***P < 0.001

In order to further investigate the effects of CACYBP knockdown on PC cell migration, transwell assay and wound-healing assay were conducted in this study. The results of wound-healing assay showed that the migration rate of LNCaP cells was obviously decreased by 44% in shCACYBP group compared with shCtrl group after 48 h (*P* < 0.05). Moreover, the migration rate of DU145 cells in shCACYBP group was 49% lower than that in the shCtrl group during 0–24 h (Fig. 4A) (*P* < 0.01). Consistently, the migratory cells per field of LNCaP and DU145 cells were decreased in shCACYBP group compared with shCtrl group based on transwell assay (*P* < 0.001) (Fig. 4B). In short, these results suggested that CACYBP knockdown could inhibit cell migration of PC cells in vitro.

### Knockdown of CACYBP suppressed tumor growth of PC in vivo

**Fig. 5:**
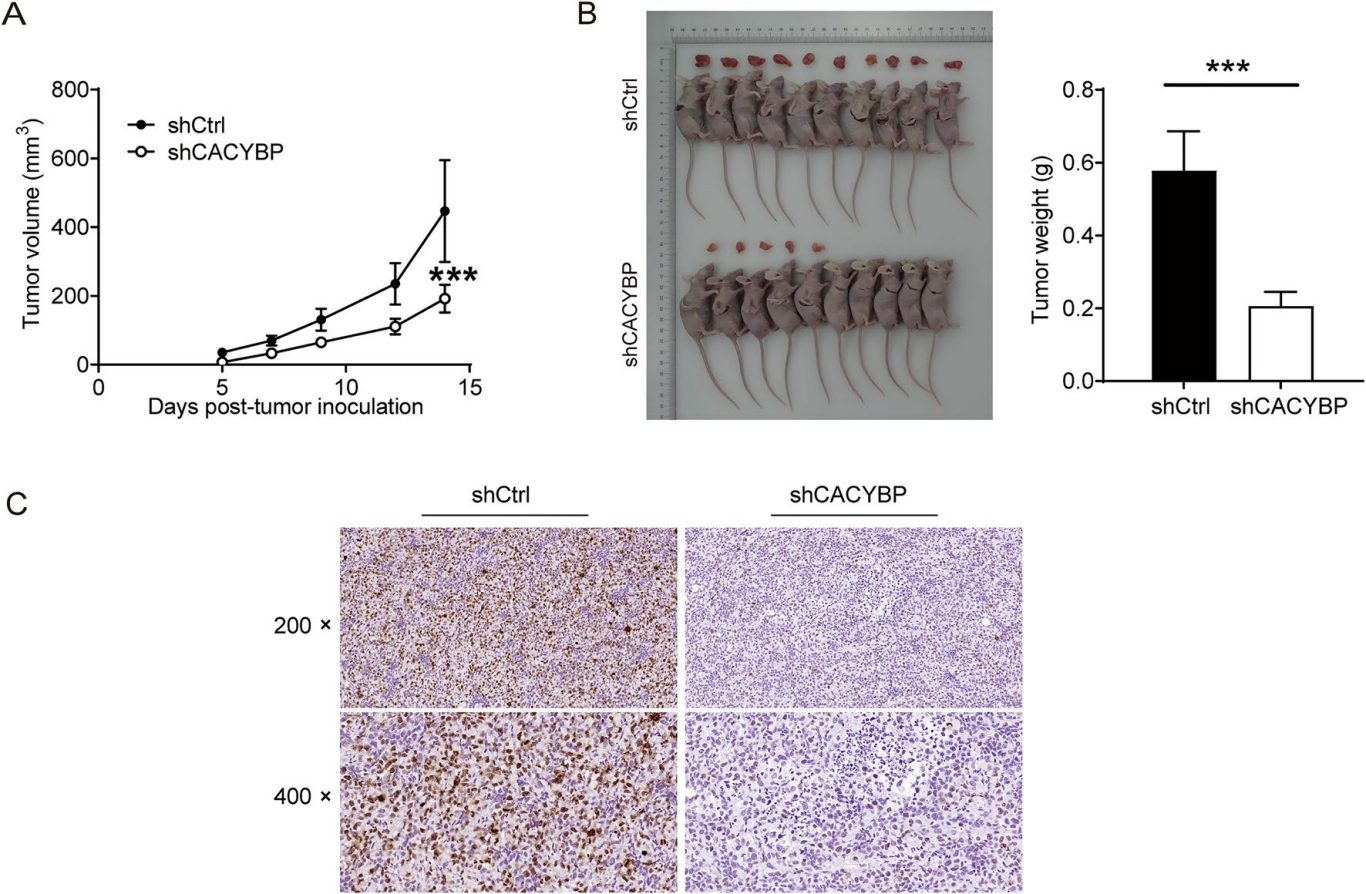
Knockdown of CACYBP suppressed tumor growth of PC in vivo. **A** The average volume of tumors in shCtrl group and shCACYBP group was measured after post-injection. **B** Images of mice and tumors in the shCtrl and shCACYBP groups. The average weight of tumors in shCtrl group and shCACYBP group was measured. **C** The expression of Ki67 expression is difference between in shCtrl and shCACYBP group, determined by IHC staining. ***P < 0.001

A mice xenograft model was established by subcutaneous injection of DU145 cells with or without CACYBP knockdown to verify the role of CACYBP in PC in vivo. The dimension of tumor size was measured from 7 days after inoculation. As shown in Fig. 5A, the tumor volume in the shCACYBP group was smaller than that in the shCtrl group (*P* < 0.001). Furthermore, CACYBP knockdown inhibited the tumors growth of PC could be directly affirmed by observing the removed tumors. The weight of removed tumors was significantly decreased in shCACYBP group (*P* < 0.001, Fig. 5B). Additionally, IHC staining further demonstrated that the expression of Ki67, a marker of cell proliferation, was lower in the shCACYBP group than the shCtrl group (*P* < 0.05) (Fig. 5C). These results confirmed the effect of CACYBP in maintaining the tumor growth of PC.

### Mechanism exploration of CACYBP in the progression of PC

**Fig. 6:**
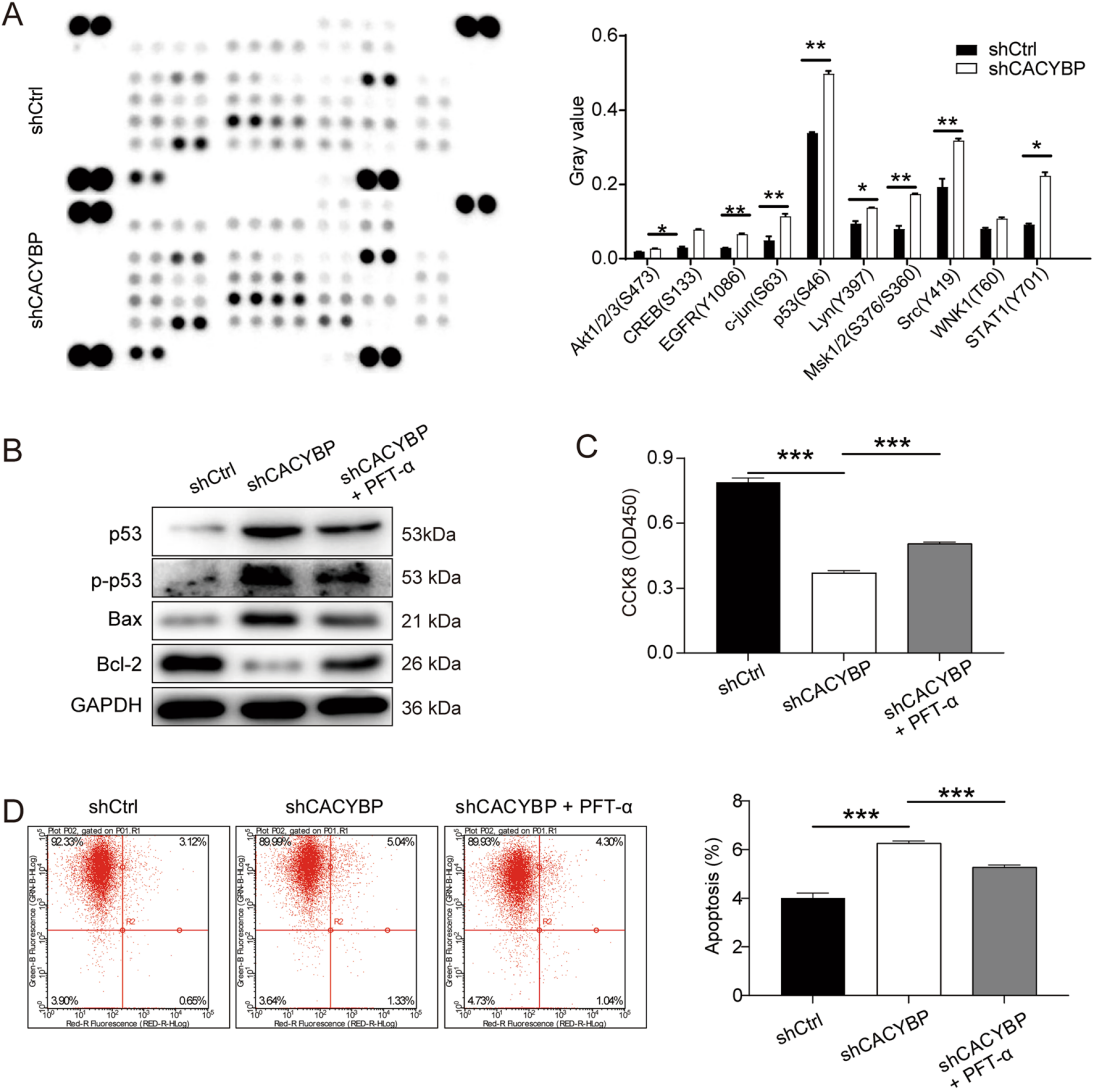
Mechanism exploration of CACYBP in the progression of PC. **A** Human phospho-kinase array was utilized to identify the phospho-kinase expression in DU145 cells with or without CACYBP knockdown. **B** Western blotting detected the expression level of related proteins after knockdown of CACYBP in DU145 cells. **C**, **D** Effect of addition of PFTα on the viability of CACYBP knocked down DU145 cells was investigated by CKK8 assay and flow cytometry. (*n* ≥ 3). **P < 0.01, ***P < 0.001

Finally, mechanism exploration of CACYBP in the progression of PC was performed using the human phospho-kinase array. As compared with the shCtrl group, the expression levels of CREB, c-jun, p53, Msk1/2 and STAT1 were significantly up-regulated in the shCACYBP group (Fig. 6A). Western blotting analysis was used to detect the expression levels of tumor suppression-related genes (p53) and apoptosis-related genes (Bax, Bcl-2) in DU145 cell line. As shown in Fig. 6B, the protein levels of p53 and Bax were up-regulated in shCACYBP group, while the protein level of Bcl-2 was significantly reduced. p53 is a tumor suppressor protein that plays an important role in the regulation of cell proliferation (Wang et al. 2021a, b, c). In this study, our results indicated that the addition of PFTα could effectively alleviate the effects of CACYBP knockdown on the cell proliferation and cell apoptosis (*P* < 0.001) (Fig. 6C, D). Combined with the present results, we could speculate that the inhibitory effects of CACYBP knockdown on development and progression of PC may be related to the p53.

## Discussion

PC, a multifactorial disease, originated in epithelial tissues with glandular organization (Davidsson et al. 2018). Although several men were diagnosed at the early stage and received treatment, most patients were diagnosed with locally advanced or metastatic disease due to many factors (Mukherji et al. 2020; Rosellini et al. 2021). Unfortunately, traditional therapies in the past have failed to meet the needs of patients with PC and even led to serious adverse reactions (Galletti et al. 2017; Nevedomskaya et al. 2018; Ruiz de Porras et al. 2021). In recent years, advances in molecular biology technology have led to the innovation of cancer treatment environment. Targeted therapy has become a promising treatment strategy for PC patients (Wang et al. 2021a, b, c). For example, ladinin-1 (LAD1) may serve as a potential prognostic factor in PC patients (Li et al. 2021). BMH-21 was considered as a new potential molecule for treatment-resistant prostate cancer (Low et al. 2019). Although the mechanism exploration of PC has made great progress, an effective molecular target for PC therapy was still needed to be discovered.

CACYBP is a multi-ligand protein implicated in the progression of various human cancers. It was reported that CacyBP/SIP was significantly increased in pancreatic cancer (Chen et al. 2011) and bladder cancer (Zheng and Chen 2021). On the contrary, some reports found that CACYBP expression was reduced in gastric cancer and renal cancer (Ning et al. 2007; Sun et al. 2007). Conflicting findings suggested that the role of CACYBP may vary depending on the cell types. Notably, our immunohistochemistry results showed that CACYBP was frequently overexpressed in PC. In addition, the expression of CACYBP was positively correlated with the pathological grade of PC patients. Interestingly, CACYBP could inhibit the proliferation of gastric cancer (Ning et al. 2007) and renal cancer cells (Sun et al. 2007). Inversely, CACYBP could promote tumor progression by regulating apoptosis, cell proliferation and invasion and arresting the cell cycle in many cancers, such as hepatocellular carcinoma (HCC) (Lian et al. 2019), colon cancer (Zhai et al. 2017) and osteosarcoma (Zhao et al. 2020). Obviously, the role of CACYBP in cancers is controversial. In the present study, our results showed that cell apoptosis was substantially promoted via knockdown of CACYBP in PC cells, while the proliferation and migration of PC were inhibited. In vivo experiment further verified the inhibitory effect of CACYBP knockdown on tumor growth of PC. All of these results suggested that CACYBP could contribute to the progression of PC.

Protein phosphorylation/dephosphorylation participates in regulation of various physiological processes such as gene expression and regulation, cell proliferation, differentiation, cell transformation and apoptosis (Wang et al. 2021a, b, c). In this study, our results indicated that CACYBP knockdown enhanced the phosphorylation of CREB, c-jun, p53, Msk1/2 and STAT1. We inferred that these proteins may be involved in the functions of tumor cells and play an important role in PC. p53, as a typical tumor suppressor protein, has been reported to perform effects by participating in DNA repair, cell cycle progression, senescence and apoptosis (Wang et al. 2021a, b, c). In this study, we initially found that p53 was involved in the process of CACYBP knockdown inhibiting the malignant progression of PC. Our present study showed that the phosphorylation of p53 was significantly increased after CACYBP knockdown. In addition, the addition of p53 inhibitor could effectively alleviate the inhibitory effect of CACYBP knockdown on cell activity. These results strongly support that p53 play a vital role in the regulation of CACYBP on the PC progression. However, the precise mechanism between CACYBP and p53 has not been fully clarified, which was the focus of our continuous attention and exploration in the later period.

In summary, CACYBP was highly expressed in PC and was positively correlated with the pathological grade of PC patients. CACYBP knockdown inhibited the proliferation and migration of PC cells in vitro and suppressed the tumor growth in vivo. Our study provided novel evidence that CACYBP contributed to the development and progression of PC through p53, which may be a novel alternative therapeutic target for PC treatment.

## Data Availability

All data generated or analyzed during this study are included in this published article.

## References

[CR1] Chen X, Mo P, Li X et al (2011) CacyBP/SIP protein promotes proliferation and G1/S transition of human pancreatic cancer cells. Mol Carcinog 50(10):804–810. 10.1002/mc.20737 21268134 10.1002/mc.20737

[CR2] Czerwińska M, Bilewicz A, Kruszewski M et al (2020) Targeted radionuclide therapy of prostate cancer-from basic research to clinical perspectives. Molecules (Basel, Switzerland). 10.3390/molecules25071743 32290196 10.3390/molecules25071743 PMC7181060

[CR3] Davidsson S, Andren O, Ohlson A et al (2018) FOXP3 regulatory T cells in normal prostate tissue, postatrophic hyperplasia, prostatic intraepithelial neoplasia, and tumor histological lesions in men with and without prostate cancer. Prostate 78(1):40–47. 10.1002/pros.23442 29105795 10.1002/pros.23442 PMC5725695

[CR4] Evans A (2018) Treatment effects in prostate cancer. Mod Pathol 31:S110–S121. 10.1038/modpathol.2017.158 29297495 10.1038/modpathol.2017.158

[CR5] Feng Q, Xu D, Zhou M et al (2021) CDC42EP3 promotes colorectal cancer through regulating cell proliferation, cell apoptosis and cell migration. Cancer Cell Int 21(1):169. 10.1186/s12935-021-01845-8 33726765 10.1186/s12935-021-01845-8 PMC7962261

[CR6] Galletti G, Leach B, Lam L et al (2017) Mechanisms of resistance to systemic therapy in metastatic castration-resistant prostate cancer. Cancer Treat Rev 57:16–27. 10.1016/j.ctrv.2017.04.008 28527407 10.1016/j.ctrv.2017.04.008

[CR7] Grüllich C, Nößner E, Pfister D et al (2020) Targeted molecular therapy and immunotherapy for prostate cancer. Der Urologe Ausg a 59(6):687–694. 10.1007/s00120-020-01198-6 32303774 10.1007/s00120-020-01198-6

[CR8] Li J, Wang Z, Tie C (2021) High expression of ladinin-1 (LAD1) predicts adverse outcomes: a new candidate docetaxel resistance gene for prostatic cancer (PCa). Bioengineered 12(1):5749–5759. 10.1080/21655979.2021.1968647 34516317 10.1080/21655979.2021.1968647 PMC8806705

[CR9] Lian Y, Huang Y, Zhang Y et al (2019) CACYBP enhances cytoplasmic retention of P27 to promote hepatocellular carcinoma progression in the absence of RNF41 mediated degradation. Theranostics 9(26):8392–8408. 10.7150/thno.36838 31754404 10.7150/thno.36838 PMC6857042

[CR10] Low J, Sirajuddin P, Moubarek M et al (2019) Effective targeting of RNA polymerase I in treatment-resistant prostate cancer. Prostate 79(16):1837–1851. 10.1002/pros.23909 31524299 10.1002/pros.23909 PMC7025478

[CR11] Mehra C, Chung J, He Y et al (2021) CdGAP promotes prostate cancer metastasis by regulating epithelial-to-mesenchymal transition, cell cycle progression, and apoptosis. Commun Biol 4(1):1042. 10.1038/s42003-021-02520-4 34493786 10.1038/s42003-021-02520-4 PMC8423782

[CR12] Mukherji D, Youssef B, Dagher C et al (2020) Management of patients with high-risk and advanced prostate cancer in the Middle East: resource-stratified consensus recommendations. World J Urol 38(3):681–693. 10.1007/s00345-019-02872-x 31297628 10.1007/s00345-019-02872-x PMC7064460

[CR13] Nevedomskaya E, Baumgart S, Haendler B (2018) Recent advances in prostate cancer treatment and drug discovery. Int J Mol Sci. 10.3390/ijms19051359 29734647 10.3390/ijms19051359 PMC5983695

[CR14] Nie F, Yu X, Wang X et al (2010) Down-regulation of CacyBP is associated with poor prognosis and the effects on COX-2 expression in breast cancer. Int J Oncol 37(5):1261–1269. 10.3892/ijo_00000777 20878073 10.3892/ijo_00000777

[CR15] Ning X, Sun S, Hong L et al (2007) Calcyclin-binding protein inhibits proliferation, tumorigenicity, and invasion of gastric cancer. Mol Cancer Res MCR 5(12):1254–1262. 10.1158/1541-7786.Mcr-06-0426 18171983 10.1158/1541-7786.MCR-06-0426

[CR16] Ratta R, Guida A, Scotté F et al (2020) PARP inhibitors as a new therapeutic option in metastatic prostate cancer: a systematic review. Prostate Cancer Prostatic Dis 23(4):549–560. 10.1038/s41391-020-0233-3 32367009 10.1038/s41391-020-0233-3

[CR17] Rosellini M, Santoni M, Mollica V et al (2021) Treating prostate cancer by antibody-drug conjugates. Int J Mol Sci. 10.3390/ijms22041551 33557050 10.3390/ijms22041551 PMC7913806

[CR18] Ruiz de Porras V, Font A, Aytes A (2021) Chemotherapy in metastatic castration-resistant prostate cancer: current scenario and future perspectives. Cancer Lett. 10.1016/j.canlet.2021.08.033 34517086 10.1016/j.canlet.2021.08.033

[CR19] Sun S, Ning X, Liu J et al (2007) Overexpressed CacyBP/SIP leads to the suppression of growth in renal cell carcinoma. Biochem Biophys Res Commun 356(4):864–871. 10.1016/j.bbrc.2007.03.080 17400182 10.1016/j.bbrc.2007.03.080

[CR20] Wang H, Zhao J, Yang J et al (2021a) PICT1 is critical for regulating the Rps27a-Mdm2-p53 pathway by microtubule polymerization inhibitor against cervical cancer. Biochim Biophys Acta 1868(10):119084. 10.1016/j.bbamcr.2021.119084 10.1016/j.bbamcr.2021.119084 34166715

[CR21] Wang L, Zhang L, Gong X et al (2021b) PP-1β and PP-2Aα modulate cAMP response element-binding protein (CREB) functions in aging control and stress response through de-regulation of αB-crystallin gene and p300–p53 signaling axis. Aging Cell. 10.1111/acel.13458 34425033 10.1111/acel.13458 PMC8441381

[CR22] Wang Z, Zhi K, Ding Z et al (2021c) Emergence in protein derived nanomedicine as anticancer therapeutics: more than a tour de force. Semin Cancer Biol 69:77–90. 10.1016/j.semcancer.2019.11.012 31962173 10.1016/j.semcancer.2019.11.012

[CR23] Yadav S, Stockert J, Hackert V et al (2018) Intratumor heterogeneity in prostate cancer. Urol Oncol 36(8):349–360. 10.1016/j.urolonc.2018.05.008 29887240 10.1016/j.urolonc.2018.05.008

[CR24] Zhai H, Shi Y, Chen X et al (2017) CacyBP/SIP promotes the proliferation of colon cancer cells. PLoS ONE 12(2):e0169959. 10.1371/journal.pone.0169959 28196083 10.1371/journal.pone.0169959 PMC5308830

[CR25] Zhao M, Zhang R, Qi D et al (2020) CacyBP/SIP promotes tumor progression by regulating apoptosis and arresting the cell cycle in osteosarcoma. Exp Ther Med 20(2):1397–1404. 10.3892/etm.2020.8843 32742374 10.3892/etm.2020.8843 PMC7388306

[CR26] Zheng H, Chen C (2021) Downregulation of CacyBP by CRISPR/dCas9-KRAB Prevents bladder cancer progression. Front Mol Biosci 8:692941. 10.3389/fmolb.2021.692941 34179100 10.3389/fmolb.2021.692941 PMC8226165

